# DMTs and Covid‐19 severity in MS: a pooled analysis from Italy and France

**DOI:** 10.1002/acn3.51408

**Published:** 2021-07-07

**Authors:** Maria Pia Sormani, Marco Salvetti, Pierre Labauge, Irene Schiavetti, Helene Zephir, Luca Carmisciano, Caroline Bensa, Nicola De Rossi, Jean Pelletier, Cinzia Cordioli, Sandra Vukusic, Lucia Moiola, Philippe Kerschen, Marta Radaelli, Marie Théaudin, Paolo Immovilli, Olivier Casez, Marco Capobianco, Jonathan Ciron, Maria Trojano, Bruno Stankoff, Alain Créange, Gioacchino Tedeschi, Pierre Clavelou, Giancarlo Comi, Eric Thouvenot, Mario Alberto Battaglia, Thibault Moreau, Francesco Patti, Jérôme De Sèze, Celine Louapre, the Musc‐19, the Covisep study groups

**Affiliations:** ^1^ Department of Health Sciences University of Genova Genova Italy; ^2^ IRCCS Ospedale Policlinico San Martino Genoa Italy; ^3^ Department of Neuroscience Mental Health and Sensory Organs Sapienza University of Rome Rome Italy; ^4^ Unit of Neurology IRCCS Neuromed Pozzilli Italy; ^5^ Department of Neurology CHU de Montpellier Montpellier France; ^6^ Department of Neurology U 1172 CRC‐SEP University Hospital of Lille Lille France; ^7^ Department of Neurology Hôpital Fondation Adolphe de Rothschild Paris France; ^8^ Centro Sclerosi Multipla ASST Spedali Civili di Brescia Montichiari Italy; ^9^ Department of Neurology Aix Marseille Univ APHM, Hôpital de la Timone Pôle de Neurosciences Cliniques Marseille 13005 France; ^10^ Service de Neurologie, sclérose en plaques, pathologies de la myéline et neuro‐inflammation Hospices Civils de Lyon Hôpital Neurologique Bron France; ^11^ Department of Neurology Multiple Sclerosis Center IRCCS Ospedale San Raffaele Milan Italy; ^12^ Centre Hospitalier de Luxembourg Luxembourg City Luxembourg; ^13^ Department of Neurology and Multiple Sclerosis Center ASST “Papa Giovanni XXIII” Bergamo Italy; ^14^ Division of Neurology Department of Clinical Neurosciences Lausanne University Hospital and University of Lausanne Lausanne Switzerland; ^15^ Multiple Sclerosis Center Ospedale Guglielmo da Saliceto Piacenza Italy; ^16^ Department of Neurology University Hospital Grenoble Alpes Neuro Inflammatory Unit Grenoble France; ^17^ Department of Neurology Regional Referral Multiple Sclerosis Centre University Hospital San Luigi Orbassano (Torino) Italy; ^18^ Department of Neurology CHU de Toulouse CRC‐SEP Toulouse France; ^19^ Department of Basic Medical Sciences Neurosciences and Sense Organs University of Bari Bari Italy; ^20^ Sorbonne University Paris Brain Institute ICM Pitié Salpêtrière Hospital Inserm UMR S 1127 CNRS UMR 7225 Paris France; ^21^ Neurology Department St Antoine Hospital APHP Paris France; ^22^ Service de Neurologie and CRC SEP APHP Groupe Hospitalier Henri Mondor UPEC Université Créteil France; ^23^ Department of Advanced Medical and Surgical Sciences University of Campania Napoli Italy; ^24^ University of Clermont Auvergne CHU de Clermont‐Ferrand Inserm Neuro‐Dol Clermont‐Ferrand France; ^25^ Institute of Experimental Neurology IRCCS Ospedale San Raffaele Milano Italy; ^26^ Department of Neurology Nîmes University Hospital Nîmes France; ^27^ Institute of Functional Genomics University of Montpellier CNRS INSERM Montpellier France; ^28^ Research Department Italian Multiple Sclerosis Foundation Genoa Italy; ^29^ Department of Life Sciences University of Siena Siena Italy; ^30^ Department of Neurology University hospital of Dijon EA4184 Dijon France; ^31^ Department of Medical and Surgical Sciences and Advanced Technologies GF Ingrassia University of Catania Catania Italy; ^32^ Centro Sclerosi Multipla Policlinico Catania University of Catania Catania Italy; ^33^ Department of Neurology CIC INSERM 1434 CHU de Strasbourg Strasbourg France; ^34^ Sorbonne University Paris Brain Institute ICM, Assistance Publique Hôpitaux de Paris APHP Hôpital de la Pitié‐Salpêtrière Inserm CNRS CIC Neuroscience Paris France

## Abstract

We evaluated the effect of DMTs on Covid‐19 severity in patients with MS, with a pooled‐analysis of two large cohorts from Italy and France. The association of baseline characteristics and DMTs with Covid‐19 severity was assessed by multivariate ordinal‐logistic models and pooled by a fixed‐effect meta‐analysis. 1066 patients with MS from Italy and 721 from France were included. In the multivariate model, anti‐CD20 therapies were significantly associated (OR = 2.05, 95%CI = 1.39–3.02, *p* < 0.001) with Covid‐19 severity, whereas interferon indicated a decreased risk (OR = 0.42, 95%CI = 0.18–0.99, *p* = 0.047). This pooled‐analysis confirms an increased risk of severe Covid‐19 in patients on anti‐CD20 therapies and supports the protective role of interferon.

## Introduction

Previous studies have reported data on Covid‐19 severity in persons with multiple sclerosis (PwMS) treated with disease‐modifying therapies (DMTs). In the Italian study,^1^ an increased risk of severe course of Covid‐19 was noted in PwMS treated with anti‐CD20 therapies and with recent use of methylprednisolone; a slight reduction of risk was observed with interferon use. The French study,^2^ including fewer patients than the Italian study, was not able to detect associations between any DMT and Covid‐19 severity, possibly due to lack of statistical power. In the US study,^3^ an increased risk associated with recent use of methylprednisolone was confirmed and rituximab was associated with more severe outcomes. Other smaller series have provided mixed results and indications.^4^, ^5^ Moreover, during the first wave of the pandemic, it was not possible to test all symptomatic patients, so the reported cohorts are a mix of suspected and confirmed Covid‐19 cases.^1^, ^2^, ^3^

Due to the relevance that these results may have for patients’ care during the pandemic, it is important to rely on well‐established data. Here we present the results of a follow‐up collection of data that extends into the “second wave” of the pandemic, pooling results on the effect of risk factors and DMTs on Covid‐19 severity using only confirmed cases from the French and Italian updated cohorts.

## Methods

Data of PwMS with suspected or confirmed Covid‐19 were retrospectively collected at a national level in Italy and France. Details on data collection and inclusion criteria were previously reported.^1^, ^2^ Only patients with a confirmed Covid‐19 diagnosis with a positive test (RT‐PCR on nasal and pharyngeal swabs) for SARS‐CoV‐2 or a positive serological test for anti‐SARS COV2 antibodies, and with complete follow‐up to death or recovery, were included in this analysis.

All analyses were conducted independently on the two datasets, after harmonizing the baseline variable coding and the definition of Covid‐19 severity. Degree of Covid‐19 severity was defined by three levels: (1) mild disease not requiring hospitalization nor ventilation; (2) hospitalization or need for ventilation; (3) ICU or death. The association of baseline characteristics and MS therapies with Covid‐19 severity was assessed by multivariate ordinal logistic models and pooled by a fixed effect meta‐analysis weighted by the inverse of the variance.

The multivariate models were adjusted by age, sex, EDSS, progressive MS course, presence of comorbidities, and recent methylprednisolone use, all variables previously identified as associated with Covid‐19 severity.^1^, ^2^ Italian data were also stratified by geographical area. Since EDSS and progressive MS course were highly associated, we ran two multivariate models including EDSS or progressive MS course separately. A heterogeneity analysis was run before deciding how to group DMTs and, excluding Interferon and anti‐CD20, the effects of other drugs did not reach a significant level of heterogeneity versus the others (in both datasets). Therefore, we focused this meta‐analysis on anti‐CD20, Interferon, other drugs, and no therapy (reference category).

## Results

Data from 1735 pwMS from Italy and 1031 pwMS from France presenting symptoms of Covid‐19 were collected. Of these, 1066 (64%) from Italy and 721 (70%) from France had confirmed Covid‐19 and were included in the meta‐analysis. Baseline demographic and clinical characteristics of the two cohorts are reported in Table 1. The two cohorts differed for some characteristics: in Italy versus France, there were less females (68% vs. 74%), more obese subjects (12% vs. 8%), and a lower proportion of subjects with secondary progressive MS (SPMS) (9.6% vs. 15.1%). The DMTs distribution was heterogeneous, with a higher proportion of pwMS in Italy versus France treated with dimethyl‐fumarate, natalizumab, interferon, cladribine, and azathioprine and a lower proportion of untreated patients and patients treated with ocrelizumab and rituximab (Table 1).

**Table 1:** Baseline demographic and clinical characteristics of the included patients.

Characteristic	Italy (*N* = 1066)	France (*N* = 721)	*P*
Age—mean (SD)	43.7 (12.3)	44.9 (13.4)	0.06
Female sex—no. (%)	724 (67.9)	534 (74.1)	0.005
BMI >30—no. (%)	124 (11.6)	56 (7.8)	0.008
Comorbidities—no. (%)	216 (20.3)	171 (23.7)	0.08
MS phenotype—no. (%)			<0.001*
Primary progressive	45 (4.2)	42 (5.8)	0.12
Relapsing remitting or CIS	918 (86.2)	567 (78.7)	0.001
Secondary progressive	102 (9.6)	109 (15.1)	<0.001
Missing data	1 (0.1)	3 (0.4)	
MS disease duration—Median (IQR)	8.6 (3.5–14.9)	10.8 (4.6–18.9)	<0.001
EDSS—Median (IQR)	2 (1–3.5)	2 (1–4)	0.15
MS treatment—no. (%)			<0.001*
Dimethyl fumarate	192 (18)	93 (12.9)	0.004
Fingolimod	128 (12)	85 (11.8)	0.88
Ocrelizumab	106 (10)	94 (13.0)	0.04
Natalizumab	152 (14)	74 (10.3)	0.01
Interferon	112 (11)	39 (5.4)	<0.001
Glatiramer‐acetate	76 (7)	55 (7.6)	0.69
Teriflunomide	81 (8)	66 (9.1)	0.24
Alemtuzumab	3 (0.3)	0	0.27
Cladribine	18 (2)	3 (0.4)	0.01
Azathioprine	13 (1)	2 (0.3)	0.04
Rituximab	20 (2)	34 (4.7)	<0.001
Methotrexate	2 (0.2)	7 (1.0)	0.02
Other	15 (1)	10 (1.4)	0.97
None	148 (14)	159 (22.1)	<0.001
Previous methylprednisolone—no. (%)	23 (2.2)	36 (5.0)	0.001

SD, Standard deviation; IQR, Inter‐quartile range.

* Test for heterogeneity.

Table 2 reports cohort characteristics according to DMT and Covid‐19 outcomes. Severity outcome was hospitalization/ventilation in 123 Italian patients (11.5%) and in 92 French patients (12.8%), and ICU/death in 27 Italian patients (2.5%) and in 19 French patients (2.7%). Seventeen Italian subjects (1.6%) and 12 French subjects (1.7%) died. In the Italian cohort, 11 pwMS had a progressive disease course, and eight were untreated. In the French cohort, nine subjects were in a progressive disease phase, and eight were untreated.

**Table 2:** Baseline characteristics and outcomes of the Italian (*n* = 1066) and the French (*n* = 721) cohorts according to disease‐modifying therapies.

Characteristic	No therapy		Interferon		Anti‐CD20		Other DMTs	
	Italy	France	Italy	France	Italy	France	Italy	France
*n* (%)	148 (13.8)	159 (22.1)	112 (10.4)	39 (5.4)	126 (11.8)	128 (17.8)	680 (64)	395 (54.8)
Age mean (SD)	51 (14)	51.0 (15.4)	44 (10)	42.1 (12.7)	44 (11)	45.3 (11.4)	42 (12)	42.6 (12.5)
Female sex no. (%)	92 (62.6)	120 (75.4)	80 (72.1)	33 (84.6)	85 (67.5)	93 (72.6)	467 (68.5)	288 (72.9)
BMI > 30 no. (%)	21 (14.3)	16 (10.0)	11 (9.9)	2 (5.1)	22 (17.5)	14 (10.9)	70 (10.3)	24 (6.1)
Comorbidities no. (%)	44 (29.9)	48 (30.2)	26 (23.3)	10 (25.6)	27 (21.4)	30 (23.4)	119 (17.4)	83 (21.0)
Progressive MS no. (%)	62 (42)	67 (42.1)	3 (2.7)	1 (2.6)	41 (32.5)	49 (38.3)	45 (6.6)	34 (8.6)
Disease duration median (IQR)	13 (5–22)	15 (4–25)	10 (5–15)	11 (6–14)	8 (4–15)	11 (7–16)	8 (3–14)	9 (4–18)
EDSS median (IQR)	3.5 (1.5–6.5)	2.5 (1–6.5)	1.5 (1–2)	1.25 (1–2)	4 (2–6)	4 (2–6)	1.5 (1–3)	1.5 (1–3)
Methyl‐pred no. (%)	6 (4.1)	12 (7.5)	1 (0.9)	0 (0)	3 (2.4)	11 (8.6)	13 (1.9)	13 (3.3)
Covid‐19 severity^1^ no (%)								
Mild	100 (68)	118 (74.2)	107 (96.4)	37 (94.9)	94 (74.6)	94 (73.4)	615 (90.2)	361 (91.4)
Hospitalized/ventilation	35 (23.8)	33 (20.8)	4 (3.6)	2 (5.1)	28 (22.2)	27 (21.1)	56 (8.2)	30 (7.6)
Intensive care unit	8 (5.4)	0 (0)	0 (0)	0 (0)	3 (2.4)	5 (3.9)	8 (1.2)	2 (0.5)
Death	8 (5.4)	8 (5.0)	0 (0)	0 (0)	3 (2.4)	2 (1.6)	6 (0.9)	2 (0.5)

DMTs, Disease‐modifying therapies; SD, Standard deviation; IQR, Inter‐quartile range; MS, Multiple Sclerosis; Methyl‐pred, Methylprednisolone.

^1^ The numbers do not sum up to the total since some patients have multiple outcomes.

**Figure 1:**
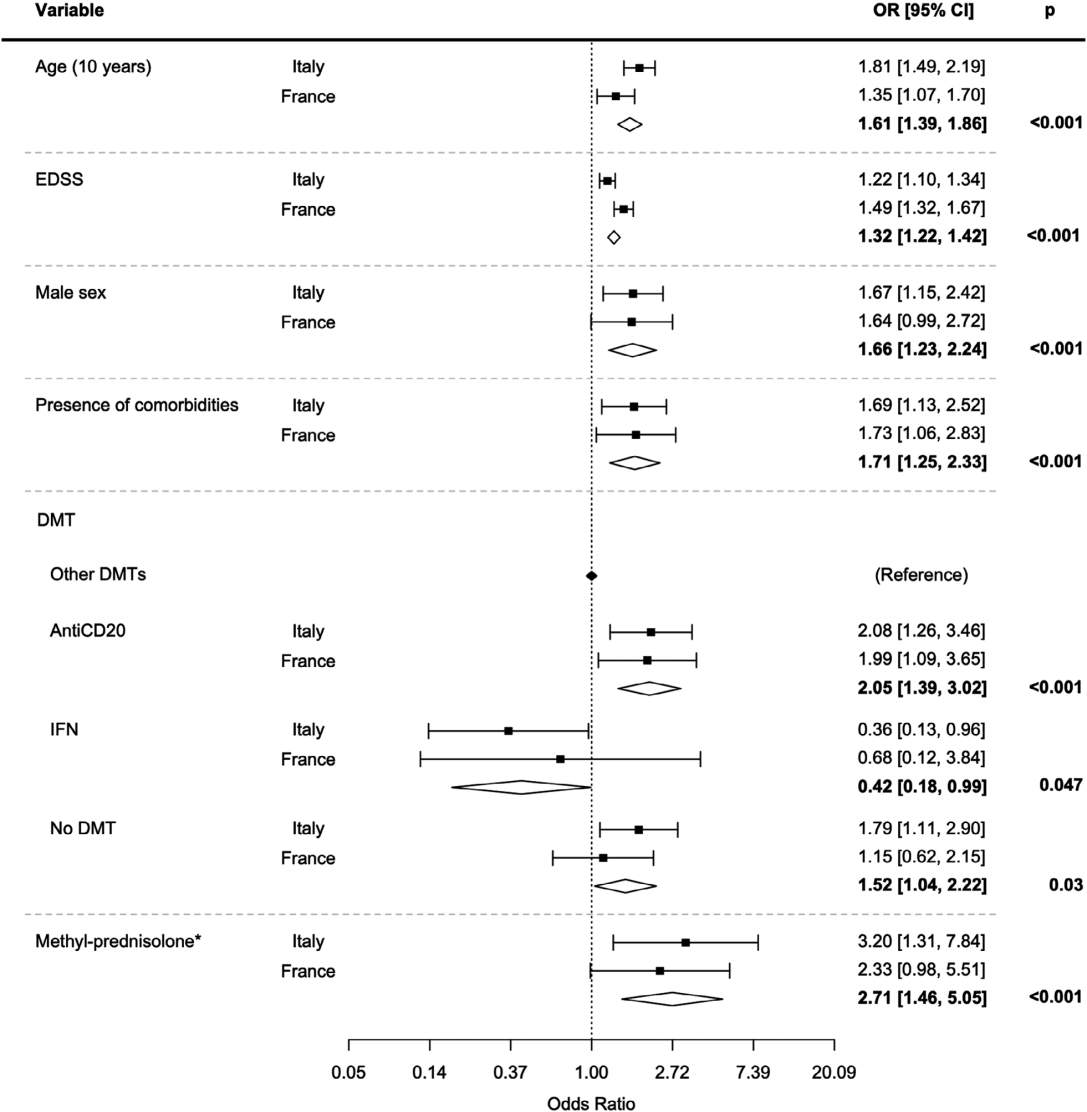
Fixed effect meta‐analysis (inverse of variance weighting) including EDSS of multivariate ordinal logistic models investigating the association between Covid‐19 severity and PwMS characteristics.

Age, male sex, EDSS, and comorbidities were all confirmed as risk factors for severe Covid‐19 (Figure 1). After adjusting for age, sex, EDSS, comorbidities, and recent methylprednisolone use, treatment with an anti‐CD20 agent (ocrelizumab or rituximab) was significantly associated (OR = 2.05,95%CI = 1.39–3.02, *p* < 0.001) with an increased risk for severe Covid‐19 versus other therapies, whereas the use of interferon was associated with a decreased risk (OR = 0.42, 95%CI = 0.18–0.99, *p* = 0.047). Recent use (<1 month) of methylprednisolone was also associated with a poorer outcome (OR = 2.71, 95%CI = 1.46–5.05, *p* < 0.001). These results were confirmed when including progressive MS instead of EDSS in the model: the OR for anti‐CD20 versus other therapies was 2.60 (95%CI = 1.79–3.77, *p* < 0.001), the OR for interferon was 0.38 (95%CI = 0.17–0.85, *p* = 0.02) and the OR for recent use of methylprednisolone was 3.11 (95%CI = 1.72–5.63, *p* = 0.001).

Both in Italy and France, the number of patients on rituximab was low (*n* = 20 in Italy and *n* = 34 in France). However, separating the effect of anti‐CD20 agents into rituximab and ocrelizumab, it was possible to detect a higher risk for rituximab (OR_Italy_ = 3.78, OR_France_ = 2.56, pooled OR = 3.04 (95%CI = 1.63, 5.67, *p* < 0.001)) versus other therapies. The effect of ocrelizumab alone was also significant (OR_Italy_ = 1.79, OR_France_ = 1.73, pooled OR = 1.77 (95%CI = 1.15, 2.72, *p* < 0.001)).

We checked for interactions with sex, but we could not find and differential effect of Interferon or anti‐CD0 between males and females.

## Discussion

Overall, this study reinforces results obtained in smaller series and from the French and Italian cohorts of pwMS: age, male sex, higher EDSS, and presence of comorbidities are relevant risk factors for severe Covid‐19. It also reconciles some apparent incongruities between the two studies on the risks related to DMT use. The increased risk of severe Covid‐19 with anti‐CD20 therapies and following a recent course of methylprednisolone, detected in the previous Italian study, is confirmed by the present analysis. Furthermore, the decreased risk of severe Covid‐19 with interferon therapy, suggested by both previous studies, has also been confirmed.

The comparison of these results to the recently published US study is limited by the large discrepancy in the frequency of severe events reported in the United States and the Italy‐France registries. In the confirmed cases, while the rate of hospitalized patients (excluding those admitted to ICU or who died) is very similar (11.5% in the Italian registry and 12.8% in the French registry vs. 13.9% in the US registry^3^), ICU admission and the mortality rates are not comparable (ICU admission 0.7% and 1.0% in Italy and France vs. 5.6% in the United States; death rate 1.6% and 1.7% in Italy and France vs. 3.6% in the United States). These differences are not justified by the different lethality rates in the general population detected in these countries, that are in the opposite direction, with larger rates in Italy (3.07%) and France (2.09%) vs. the United States (1.82) (https://coronavirus.jhu.edu/map.html, accessed on March 27 2021). These differences indicate that caution is needed in comparing Italy and France with results from the United States.

The role of older age, male sex, higher EDSS, and presence of comorbidities detected in this pooled analysis is in line with the US study, as is the impact of steroid use prior to infection. A higher risk for severe Covid‐19 associated with anti‐CD20 therapies was only detected in rituximab‐treated subjects in the US registry. In both Italy and France, the number of patients on rituximab was low, but separating the effect of rituximab from ocrelizumab, a higher risk for rituximab was still detectable, whereas ocrelizumab also had a significant effect. A possible explanation of a higher risk associated with rituximab is the longer exposure to rituximab therapy, as suggested by the Italian study.^1^

With some caveats, the conclusions of this study are in accordance with current knowledge about the biology of Covid‐19. Concerning anti‐CD20 therapies, a mature B‐cell response is important for neutralizing SARS‐CoV‐2, either by preventing the virus from entering the cell or by lysing infected cells.^6^ Moreover, a larger pool of SARS‐CoV‐2‐specific, naïve B cells is associated with a better antiviral response.^7^ However, uncertainties remain about the relative impact of B cells on Covid‐19 pathophysiology compared to other lymphocyte subsets.^8^ In particular, it is possible that T cells, a robust innate immune response,^9^ or the relative sparing of the IgA response in the mucosal‐associated lymphoid tissue,^10^ may variably contribute to preventing serious Covid‐19 even in the absence of high‐titer neutralizing antibodies. This may explain observations where B‐cell depletion had limited or no consequences on Covid‐19.^11^, ^12^

The increased risk of severe Covid‐19 after recent administration of high‐dose methylprednisolone is in line with well‐known immunosuppressive effects of corticosteroids. The RECOVERY^13^ trial, demonstrating the efficacy of dexamethasone in hospitalized patients with Covid‐19, does not contrast with our results. In fact, the immunosuppressive effects of methylprednisolone given prior to Covid‐19 onset plausibly intercept the phase of active viral replication with obvious negative consequences.

Finally, following some conflicting results, the prevailing opinion is now that an impaired type I interferon response correlates with Covid‐19 severity. It is therefore plausible that ongoing interferon therapy may offer some protection, at least in subjects with a defective type I interferon response.^14^, ^15^

Our findings provide a reliable reference for clinical decisions. However, due to the emergence of new viral variants, it will be important to continue monitoring the safety of immunosuppressive therapies.

## Conflicts of Interest

M.P.S. reports a grant from Roche to cover Musc‐19 data management; Roche produces ocrelizumab, which is one of the DMTs assessed in this study. The other authors have nothing to report.

## Author Contribution

Musc‐19 and COVISEP study group participants are listed in Data S1.

## Supporting information


**Data S1**. Group author list
