# Supplementary material for: DMTs and Covid‐19 severity in MS: a pooled analysis from Italy and France
Source: Ann Clin Transl Neurol. 2021 Jul 7;8(8):1738–44. doi: 10.1002/acn3.51408 (PMC8351392; doi:10.1002/acn3.51408)
Supplement: Supplementary file 1 — Data S1. Group author list [file ACN3-8-1738-s001.pdf]

Name	Affiliation	State
Abbadessa Gianmarco	Department of Advanced Medical and Surgical Sciences, University of Campania Luigi Vanvitelli, 80138 Naples, Italy;	Italy
Aguglia Umberto	Department of medical and surgical sciences, Magna Graecia University Catanzaro	Italy
Allegorico Lia	Multiple Sclerosis Centre A. Cardarelli Hospital, Naples, Italy	Italy
Allegri Rossi Beatrice Maria	Centro SM Fidenza (PR)	Italy
Alteno Anastasia	MS Center, Department of Neuroscience, City of Health and Science University Hospital of Turin, Turin, Italy	Italy
Amato Maria Pia	Università degli Studi di Firenze, Dipartimento NEUROFARBA, Firenze - IRCCS Fondazione Don Carlo Gnocchi, Firenze	Italy
Annovazzi Pietro	Centro Sclerosi Multipla Ospedale di Gallarate, ASST della Valle Olona	Italy
Antozzi Carlo	Centro Sclerosi Multipla, U.O Neurologia IV, Fondazione IRCCS Istituto Neurologico "Carlo Besta", Milano	Italy
Appendino Lucia	SC Neurologia1 Ospedale Maria Vittoria- Torino	Italy
Arena Sebastiano	Dipartimento Scienze Mediche e Chirurgiche e Tecnologie Avanzate, GF Ingrassia, Università di Catania; Centro Sclerosi Multipla Policlinico "G Rodolico"- San Marco, Università di Catania	Italy
Baione Viola	Department of Human Neurosciences, Sapienza, University of Rome	Italy
Balgera Roberto	MS Center, ASST Lecco	Italy
Barcella Valeria	USS Neuroimmunologia, ASST Papa Giovanni XXIII	Italy
Baroncini Damiano	Centro Sclerosi Multipla Ospedale di Gallarate, ASST della Valle Olona	Italy
Barrilà Caterina	ASST Rhodense	Italy
Battaglia Mario A.	1. Research Department, Italian Multiple Sclerosis Foundation, Genoa, Italy.	Italy
	2. Department of Life Sciences, University of Siena, Siena, Italy.	Italy
Bellacosa Alessandra	Centro Sclerosi Multipla, UO Neurologia, Ospedale San Giacomo, Monopoli (Bari)	Italy
Bellucci Gianmarco	Department of Neuroscience, Mental Health and Sensory Organs Sapienza University S. Andrea Hospital-site Rome	Italy
Bergamaschi Roberto	IRCCS Mondino Foundation, Pavia	Italy
Bergamaschi Valeria	AISM Rehabilitation Service Liguria	Italy
Bezzini Daiana	Department of Life Sciences, University of Siena, Siena, Italy	Italy
Biolzi Beatrice	Centro SM Fidenza (PR)	Italy
Bisecco Alvino	Centro SM, I Clinica Neurologica, AOU-Policlinico, Università della Campania "Luigi Vanvitelli"	Italy
Bonavita Simona	Department of Advanced Medical and Surgical Sciences, University of Campania Luigi Vanvitelli, 80138 Naples, Italy;	Italy
Borriello Giovanna	NCL Isituto di Neuroscienze Roma	Italy
Bosa Chiara	MS Center, Department of Neuroscience, City of Health and Science University Hospital of Turin, Turin, Italy	Italy
Bosco Antonio	Neurology Unit, Department of Medical, Surgical, and Health Sciences, Cattinara University Hospital, ASUGI, Trieste	Italy
Bovis Francesca	Department of Health Sciences, University of Genoa, Genoa, Italy.	Italy
Bozzali Marco	Neurology II, Dept of Neuroscience, University of Turin	Italy
Brambilla Laura	Centro Sclerosi Multipla, U.O Neurologia IV, Fondazione IRCCS Istituto Neurologico "Carlo Besta", Milano	Italy
Brescia Morra Vincenzo	Federico II University of Naples	Italy
Brichetto Giampaolo	AISM Rehabilitation Service Liguria	Italy
Buccafusca Maria	Centro Sclerosi Multipla UOC Neurologia e Malattie Neuromuscolari, AOU Policlinico G. Martino Messina	Italy
Bucciantini Elisabetta	Ospedale Savigliano ASLCN1	Italy
Bucello Sebastiano	Centro SM Ospedale Muscatello Augusta (ASP8 SR)	Italy
Buscarinu Maria Chiara	Department of Neuroscience, Mental Health and Sensory Organs Sapienza University S. Andrea Hospital-site Rome	Italy
Cabboi Maria Paola	UOC Neurologia- centro SM- AUSL- IRCCS RE	Italy
Calabrese Massimiliano	The Multiple Sclerosis Center of University Hospital of Verona Dept. of Neuroscience, Biomedicine and Movements	Italy
Calabria Francesca	UO Neurologia A-Azienda Ospedaliera Universitaria Integrata Verona	Italy
Caleri Francesca	Multiple Sclerosis Center, Department of Neurology - Franz Tappeiner Hospital Meran (BZ), Italy	Italy
Camilli Federico	IRCCS Istituto delle Scienze Neurologiche di Bologna, UOSI Riabilitazione Sclerosi Multipla	Italy
Caniatti Luisa Maria	Centro sclerosi Multipla azienda ospedaliera universitaria S. Anna, Ferrara	Italy
Cantello Roberto	Neurology Unit, Maggiore della Carità Hospital, Department of Translational Medicine, University of Piemonte Orientale, Novara, Italy	Italy
Capobianco Marco	SCDO Neurologia e Centro di Riferimento Regionale Sclerosi Multipla, AOU San Luigi - Orbassano (TO)	Italy
Capra Ruggero	Centro Sclerosi Multipla ASST Spedali Civili di Brescia, Ospedale di Montichiari	Italy
Capuano Rocco	Centro SM, I Clinica Neurologica, AOU-Policlinico, Università della Campania "Luigi Vanvitelli"	Italy
Carmisciano Luca	Department of Health Sciences, University of Genoa, Genoa, Italy.	Italy
Carta Patrizia	Centro Sclerosi Multipla Ospedale di Gallarate, ASST della Valle Olona	Italy
Cavalla Paola	MS Center, Department of Neuroscience, City of Health and Science University Hospital of Turin, Turin, Italy	Italy
Celani Maria Grazia	Azienda Ospedaliera di Perugia, SC di Neurofisiopatologia	Italy
Cellerino Maria	DINOGMI Universita' di Genova	Italy
Cerqua Raffaella	Clinica Neurologica Ospedali Riuniti Ancona	Italy
Chisari Clara	Dipartimento Scienze Mediche e Chirurgiche e Tecnologie Avanzate, GF Ingrassia, Università di Catania; Centro Sclerosi Multipla Policlinico "G Rodolico"- San Marco, Università di Catania	Italy
Clerici Raffaella	Centro Sclerosi Multipla U.O. Neurologia Ospedale Valduce Como	Italy
Clerico Marinella	Clinical and Biological Sciences Dept, University of Torino	Italy
Cocco Eleonora	Centro Sclerosi Multipla, ATS Sardegna/ Dpt Scienze Mediche e Sanità Publica, Università di Cagliari, Cagliari	Italy
Cola Gaia	Multiple Sclerosis Clinical and Research Unit, Department of Systems Medicine, Tor Vergata University, Rome, Italy	Italy
Comi Giancarlo	Institute of Experimental Neurology, IRCCS Ospedale San Raffaele, Milan, Italy.	Italy
Confalonieri Paolo	Centro Sclerosi Multipla, U.O Neurologia IV, Fondazione IRCCS Istituto Neurologico "Carlo Besta", Milano	Italy
Conte Antonella	1) Department of Human Neurosciences, Sapienza, University of Rome. 2) IRCCS Neuromed, Pozzilli (IS)	Italy
Conti Marta Zaffira	USS Neuroimmunologia, ASST Papa Giovanni XXIII	Italy
Cordano Christian	UCSF, San Francisco, USA	Italy
Cordera Susanna	SC Neurologia Ausl Valle D' Aosta	Italy
Cordioli Cinzia	Centro Sclerosi Multipla ASST Spedali Civili di Brescia, Ospedale di Montichiari	Italy
Corea Francesco	Neurologia, Ospedale San Giovanni, Foligno	Italy
Correale Claudio	AISM Vicenza Rehabilitation Service	Italy
Cottone Salvatore	Centro Sclerosi Multipla U.O.C. Neurologia con Stroke Unit A.R.N.A.S Civico (Palermo)	Italy
Crescenzo Francesco	The Multiple Sclerosis Center of University Hospital of Verona Dept. of Neuroscience, Biomedicine and Movements	Italy
Curti Erica	Multiple Sclerosis Centre, Department of General Medicine, Parma University Hospital, Parma	Italy
d'Ambrosio Alessandro	Centro SM, I Clinica Neurologica, AOU-Policlinico, Università della Campania "Luigi Vanvitelli"	Italy
D'Amico Emanuele	Dipartimento Scienze Mediche e Chirurgiche e Tecnologie Avanzate, GF Ingrassia, Università di Catania; Centro Sclerosi Multipla Policlinico "G Rodolico"- San Marco, Università di Catania	Italy
Danni Maura Chiara	Clinica Neurologica Ospedali Riuniti Ancona	Italy
d'Arma Alessia	IRCCS Fondazione Don Carlo Gnocchi ONLUS, Milano	Italy
Dattola Vincenzo	UOC Neurologia, Grande Ospedale Metropolitano "Bianchi Melacrino Morelli", Reggio di Calabria	Italy
de Biase Stefano	Neurology Unit, Ospedale dell'Angelo, Venezia-Mestre, Italy	Italy
De Luca Giovanna	MS Centre, Department of Clinical Neurology, SS. Annunziata,� University Hospital, Chieti, Italy	Italy
De Mercanti Stefania Federica	Clinical and Biological Sciences Dept, University of Torino	Italy
De Mitri Paolo	Emergency Department, Guglielmo da Saliceto Hospital, Piacenza, Italy	Italy
De Rossi Nicola	Centro Sclerosi Multipla ASST Spedali Civili di Brescia, Ospedale di Montichiari	Italy
De Stefano Nicola	Department of Medicine, Surgery and Neuroscience, University of Siena, Italy	Italy
Della Cava Marco	AISM Padova Rehabilitation Service	Italy
di Napoli Mario	Centro Sclerosi Multipla Rieti (Rieti)	Italy
Di Sapio Alessia	Department of Neurology, Regina Montis Regalis Hospital, Mondovì (CN)	Italy
Docimo Renato	Centro Sclerosi Multipla, Presidio Ospedaliero "San Giuseppe Moscati" “ ASL Caserta, Aversa (CE).	Italy
Dutto Anna	Ospedale Savigliano Aslcn1	Italy
Evangelista Luana	Demyelinating Disease Center, San Salvatore Hospital, L'Aquila	Italy
Fanara Salvatore	Department of Biomedicine, Neurosciences and Advanced Diagnsotics; University of Palermo	Italy
Ferraro Diana	Department of Biomedical, Metabolic and Neurosciences, University of Modena and Reggio Emilia, Modena, Italy	Italy
Ferrò Maria Teresa	Neuroimmunology, Center for Multiple Sclerosis, ASST, Crema, Italy	Italy
Filippi Massimo	1 Neuroimaging Research Unit, Institute of Experimental Neurology, Division of Neuroscience, IRCCS San Raffaele Scientific Institute, Milan, Italy	Italy
	2 Neurology Unit, IRCCS San Raffaele Scientific Institute, Milan, Italy	Italy
	3 Neurorehabilitation Unit, IRCCS San Raffaele Scientific Institute, Milan, Italy	Italy
	4 Neurophysiology Unit, IRCCS San Raffaele Scientific Institute, Milan, Italy	Italy
	5 Vita-Salute San Raffaele University, Milan, Italy	Italy
Fioretti Cristina	UO Neurologia Livorno	Italy
Fratta Mario	II Clinica Neurologica, Università della Campania Luigi Vanvitelli, Naples	Italy
Frau Jessica	Centro Sclerosi Multipla, ATS Sardegna/ Dpt Scienze Mediche e Sanità Publica, Università di Cagliari, Cagliari	Italy
Fronza Marzia	Centro Sclerosi Multipla, ATS Sardegna/ Dpt Scienze Mediche e Sanità Publica, Università di Cagliari, Cagliari	Italy
Gajofatto Alberto	Dipartimento di Neuroscienze, Biomedicina e Movimento, Università di Verona	Italy
Gallo Antonio	Centro SM, I Clinica Neurologica, AOU-Policlinico, Università della Campania "Luigi Vanvitelli"	Italy
Gallo Paolo	Multiple Sclerosis Centre of the Veneto Region (CeSMuV), University Hospital of Padua, Italy.	Italy
Gasperini Claudio	Dept Neurosciences, San Camillo Forlanini Hospital - Rome	Italy
Ghazaryan Anna	UO Neurologia	Italy
Giometto Bruno	Ospedale Santa Chiara, Trento. UO Neurologia (Trento)	Italy
Gobbin Francesca	Dipartimento di Neuroscienze, Biomedicina e Movimento, Università di Verona	Italy
Govone Flora	Department of Neurology, Regina Montis Regalis Hospital, Mondovì (CN)	Italy
Granella Franco	Unit of Neurosciences, Department of Medicine and Surgery, University of Parma, Parma & Multiple Sclerosis Centre, Department of General Medicine, Parma University Hospital, Parma	Italy
Grange Erica	Dept. of Rehabilitation, CRRF "Mons. Luigi Novarese", Moncrivello, Italy	Italy
Grasso Maria Grazia	IRCCS Fondazione Santa Lucia	Italy
Guareschi Angelica	Centro SM Fidenza (PR)	Italy
Guaschino Clara	Centro Sclerosi Multipla Ospedale di Gallarate, ASST della Valle Olona	Italy
Guerrieri Simone	Neurology Department, Multiple Sclerosis Center, San Raffaele Hospital, Milan	Italy
Guidetti Donata	Emergency Department, Guglielmo da Saliceto Hospital, Piacenza, Italy	Italy
Iaffaldano Pietro	Department of Basic Medical Sciences, Neurosciences and Sense Organs - University of Bari Aldo Moro	Italy
Ianniello Antonio	Centro SM S.Andrea Dip. Neuroscienze Umane Sapienza Roma	Italy
Iasevoli Luigi	IRCCS Fondazione Santa Lucia	Italy
Immovilli Paolo	Emergency Department, Guglielmo da Saliceto Hospital, Piacenza, Italy	Italy
Imperiale Daniele	SC Neurologia1 Ospedale Maria Vittoria- Torino	Italy
Infante Maria Teresa	Neurologia ASL 1 imperiese	Italy
Inglese Matilde	DINOGMI Universita' di Genova	Italy
Iodice Rosa	Department of Neuroscience, Reproductive Sciences and Odontostomatology, University Federico II of Naples, Naples, Italy	Italy
Iovino Aniello	Department of Advanced Biomedical Sciences, University Federico II, Naples, Italy	Italy
Konrad Giovanna	AISM Aosta Rehabilitation Service	Italy
Landi Doriana	Multiple Sclerosis Clinical and Research Unit, Department of Systems Medicine, Tor Vergata University, Rome, Italy	Italy
Lanzillo Roberta	Federico II University of Naples	Italy
Lapucci Caterina	DINOGMI Universita' di Genova	Italy
Lavorgna Luigi	Department of Advanced Medical and Surgical Sciences, University of Campania Luigi Vanvitelli, 80138 Naples, Italy;	Italy
L'Episcopo Maria Rita	Centro Sclerosi Multipla ospedale San Lazzaro (Alba, CN)	Italy
Leva Serena	Centro Sclerosi Multipla, Ospedale di Legnano, ASST OVEST MI, Italia	Italy
Liberatore Giuseppe	Neuromuscular and Neuroimmunology Service, IRCCS Humanitas Clinical and Research Institute, Rozzano, Milan, Italy	Italy
Lo Re Marianna	SCDO Neurologia e Centro di Riferimento Regionale Sclerosi Multipla, AOU San Luigi - Orbassano (TO)	Italy
Longoni Marco	Local Health Agency of Romagna, Maurizio Bufalini Hospital (Cesena) - Neurology Unit	Italy
Lopiano Leonardo	Neurology II, Dept of Neuroscience, University of Turin	Italy
Lorefice Lorena	Centro Sclerosi Multipla, ATS Sardegna/ Dpt Scienze Mediche e Sanità Publica, Università di Cagliari, Cagliari	Italy
Lucchini Matteo	Fondazione Policlinico Universitario Agostino Gemelli IRCCS - Università Cattolica del Sacro Cuore	Italy
Lus Giacomo	II Clinica Neurologica, Università della Campania Luigi Vanvitelli, Naples	Italy
Maimone Davide	Centro SM - UOC Neurologia - ARNAS Garibaldi - Catania	Italy
Malentacchi Maria	SCDO Neurologia e Centro di Riferimento Regionale Sclerosi Multipla, AOU San Luigi - Orbassano (TO)	Italy
Mallucci Giulia	IRCCS Mondino Foundation, Pavia	Italy

Malucchi Simona	SCDO Neurologia e Centro di Riferimento Regionale Sclerosi Multipla, AOU San Luigi - Orbassano (TO)	Italy
Mancinelli Chiara Rosa	Centro Sclerosi Multipla ASST Spedali Civili di Brescia, Ospedale di Montichiari	Italy
Mancinelli Luca	Local Health Agency of Romagna, Maurizio Bufalini Hospital (Cesena) - Neurology Unit	Italy
Manganotti Paolo	Neurology Unit, Department of Medical, Surgical, and Health Sciences, Cattinara University Hospital, ASUGI, Trieste	Italy
Maniscalco Giorgia Teresa	Multiple Sclerosis Centre "A. Cardarelli Hospital", Naples, Italy	Italy
	Neurological Clinic and Stroke Unit "A. Cardarelli Hospital", Naples, Italy;	Italy
Mantero Vittorio	MS Center, ASST Lecco	Italy
Marangoni Sabrina	Ospedale Santa Chiara, Trento. UO Neurologia (Trento)	Italy
Marastoni Damiano	The Multiple Sclerosis Center of University Hospital of Verona Dept. of Neuroscience, Biomedicine and Movements	Italy
Marfia Girolama Alessandra	Multiple Sclerosis Clinical and Research Unit, Department of Systems Medicine, Tor Vergata University, Rome, Italy	Italy
	Unit of Neurology, IRCCS Istituto Neurologico Mediterraneo NEUROMED, Pozzilli, IS, Italy	Italy
Marinelli Fabiana	Ospedale Fabrizio Spaziani Frosinone	Italy
	Asl Frosinone	Italy
Marti Alessandro	UOC Neurologia- centro SM Reggio Emilia- AUSL-IRCSS RE	Italy
Martinelli Boneschi Filippo	1. IRCCS Fondazione Ca' Granda Ospedale Maggiore Policlinico, Neurology Unit, Milan, Italy. Via Francesco Sforza 35, 20122	Italy
		Italy
	2. Dino Ferrari Center, Department of Pathophysiology and Transplantation, University of Milan, Milan, Italy. Via Francesco Sforza 35, 20122	Italy
Masserano Zoli Federco	AISM Padova Rehabilitation Service	Italy
Matta Francesca	Centro SM - UOC Neurologia - ARNAS Garibaldi - Catania	Italy
Mendozzi Laura	IRCCS Fondazione Don Carlo Gnocchi ONLUS, Milano	Italy
Meucci Giuseppe	UO Neurologia Livorno	Italy
Miante Silvia	Multiple Sclerosis Centre of the Veneto Region (CeSMuV), University Hospital of Padua, Italy.	Italy
Miele Giuseppina	Department of Advanced Medical and Surgical Sciences, University of Campania Luigi Vanvitelli, 80138 Naples, Italy;	Italy
Milano Eva	SC Neurologia1 Ospedale Maria Vittoria- Torino	Italy
Mirabella Massimiliano	Fondazione Policlinico Universitario Agostino Gemelli IRCCS - Università Cattolica del Sacro Cuore	Italy
Missione Rosanna	II Clinica Neurologica, Università della Campania Luigi Vanvitelli, Naples	Italy
Moccia Marcello	Federico II University of Naples	Italy
Moiola Lucia	Neurology Unit, IRCCS San Raffaele Scientific Institute, Milan, Italy	Italy
Montepietra Sara	Responsabile del Centro Sclerosi Multipla - Reggio Emilia- UOC Neurologia- AUSL-IRCSS RE	Italy
MontiBragadin Margherita	AISM Rehabilitation Service Liguria	Italy
Montini Federico	Neurology Unit, IRCCS San Raffaele Scientific Institute, Milan, Italy	Italy
Motta Roberta	AISM Rehabilitation Service Liguria	Italy
Nardone Raffaele	1. Paracelsus Medical University, Department of Neurology, Salisburgo, AU	Italy
	2. Department of Neurology - Franz Tappeiner Hospital Meran (BZ), Italy	Italy
Nicoletti Carolina Gabri	Multiple Sclerosis Clinical and Research Unit, Department of Systems Medicine, Tor Vergata University, Rome, Italy	Italy
Nobile-Orazio Eduardo	1)Neuromuscular and Neuroimmunology Service, IRCCS Humanitas Clinical and Research Institute, Rozzano, Milan, Italy	Italy
	2)Department of Medical Biotechnology and Translational Medicine, Milan University, Milan,	Italy
	Italy	Italy
Nozzolillo Agostino	Neurology Unit, IRCCS San Raffaele Scientific Institute, Milan, Italy	Italy
Onofrj Marco	Department of Neurosciences, Imaging and Clinical Sciences, University G. d'Annunzio of Chieti-Pescara, Chieti, Italy	Italy
Orlandi Riccardo	Dipartimento di Neuroscienze, Biomedicina e Movimento, Università di Verona	Italy
Palmieri Anna	UO Neurologia, Treviso	Italy
Paolicelli Damiano	Department of Basic Medical Sciences, Neurosciences and Sense Organs - University of Bari Aldo Moro	Italy
Pasquali Livia	Department of Clinical and Experimental Medicine, Neurology Unit, University of Pisa, Pisa, Italy	Italy
Pastò Luisa	Azienda Ospedaliero Universitaria Careggi, Firenze	Italy
Patti Francesco	Dipartimento Scienze Mediche e Chirurgiche e Tecnologie Avanzate, GF Ingrassia, Università di Catania; Centro Sclerosi Multipla Policlinico "G Rodolico"- San Marco, Università di Catania	Italy
Pedrazzoli Elisabetta	AISM Padova Rehabilitation Service	Italy
Perini Paola	Multiple Sclerosis Centre of the Veneto Region (CeSMuV), University Hospital of Padua, Italy.	Italy
Pesci Ilaria	Responsabile Centro SM Fidenza (PR)	Italy
Petracca Maria	Federico II University of Naples	Italy
Petrone Alfredo	Ospedale Annunziata (Cosenza)	Italy
Piantadosi Carlo	UOC Neurologia - Azienda Ospedaliera "San Giovanni-Addolorata" - Roma	Italy
Pietroboni Anna M.	1. Fondazione IRCCS Ca' Granda Ospedale Maggiore Policlinico, Milan, IT.	Italy
		Italy
	2. University of Milan, Dino Ferrari Center, Milan, IT.	Italy
Pinardi Federica	IRCCS Istituto delle Scienze Neurologiche di Bologna, UOSI Riabilitazione Sclerosi Multipla	Italy
Ponzano Marta	Department of Health Sciences, University of Genoa, Genoa, Italy.	Italy
Portaccio Emilio	Università degli Studi di Firenze, Dipartimento NEUROFARBA, Firenze	Italy
Pozzato Mattia	1. IRCCS Fondazione Ca' Granda Ospedale Maggiore Policlinico, Neurology Unit, Milan, Italy. Via Francesco Sforza 35, 20122	Italy
		Italy
	2. Dino Ferrari Center, Department of Pathophysiology and Transplantation, University of Milan, Milan, Italy. Via Francesco Sforza 35, 20122	Italy
Pozzilli Carlo	Centro SM S.Andrea Dip.Neuroscienze Umane Sapienza Roma	Italy
Prosperini Luca	Dept Neurosciences, San Camillo Forlanini Hospital - Rome	Italy
Protti Alessandra	ASST GOM NIGUARDA, DIPARTIMENTO NEUROSCIENZE	Italy
Radaelli Marta	USS Neuroimmunologia, ASST Papa Giovanni XXIII	Italy
Ragonese Paolo	Department of Biomedicine, Neurosciences and Advanced Diagnsotics; University of Palermo	Italy
Rasia Sarah	Centro Sclerosi Multipla ASST Spedali Civili di Brescia, Ospedale di Montichiari	Italy
Realmuto Sabrina	Centro Sclerosi Multipla, UOC di Neurologia e Stroke Unit, AOOR Villa Sofia-Cervello Palermo	Italy
Repice Anna	Azienda Ospedaliera Universitaria Careggi Firenze	Italy
Rigoni Eleonora	IRCCS Mondino Foundation, Pavia	Italy
Rilla Maria Teresa	Neurologia ASL 1 imperiese	Italy
Rinaldi Francesca	Multiple Sclerosis Centre of the Veneto Region (CeSMuV), University Hospital of Padua, Italy.	Italy
Romano Calogero Marcello	Centro Sclerosi Multipla, UOC di Neurologia e Stroke Unit, AOOR Villa Sofia-Cervello Palermo	Italy
Ronzoni Marco	ASST Rhodense	Italy
Rovaris Marco	IRCCS Fondazione Don Carlo Gnocchi ONLUS, Milano	Italy
Ruscica Francesca	centro SM reparto neurologia Ospedale G. Giglio, Cefalù (PA)	Italy
Sabattini Loredana	IRCCS Istituto delle Scienze Neurologiche di Bologna, UOSI Riabilitazione Sclerosi Multipla	Italy
Salemi Giuseppe	Department of Biomedicine, Neurosciences and advanced Diagnostics; University of Palermo	Italy
Salvetti Marco	1. Department of Neuroscience, Mental Health, and Sensory Organs, Sapienza University of Rome, Rome, Italy.	Italy
	2. Unit of Neurology, IRCCS Neuromed, Pozzilli, Italy.	Italy
Saraceno Lorenzo	ASST GOM NIGUARDA, DIPARTIMENTO NEUROSCIENZE	Italy
Sartori Alessia	Department of Pharmacy, Ospedale Guglielmo da Saliceto, Piacenza , Italy	Italy
Sartori Arianna	Neurology Unit, Department of Medical, Surgical, and Health Sciences, Cattinara University Hospital, ASUGI, Trieste	Italy
Sbragia Elvira	DINOGMI Universita' di Genova	Italy
Scandellari Cinzia	IRCCS Istituto delle Scienze Neurologiche di Bologna, UOSI Riabilitazione Sclerosi Multipla	Italy
Scarano Giuditta Ilaria	1. Departement of Psycology, University of Milano-Bicocca, Milan, Italy	Italy
	2. Departement of Psychology, Franz Tappeiner Hospital, Merano, Italy	Italy
Scarano Valentina	UOC Neurologia e Stroke AORN San Giuseppe Moscati Avellino	Italy
Schiavetti Irene	Department of Health Sciences, University of Genoa, Genoa, Italy.	Italy
Sessa Maria	UOC Neurologia, USS Neuroimmunologia, ASST Papa Giovanni XXIII	Italy
Sgarito Caterina	AISM Como Rehabilitation Service	Italy
Sibilia Grazia	NEUROLOGY UNIT AND MS CENTER, ASL NAPOLI 1 CENTRO, NAPLES, ITALY	Italy
Siciliano Gabriele	Department of Clinical and Experimental Medicine, Neurology Unit, University of Pisa, Pisa, Italy	Italy
Signori Alessio	Department of Health Sciences, University of Genoa, Genoa, Italy.	Italy
Signoriello Elisabetta	II Clinica Neurologica, Università della Campania Luigi Vanvitelli, Naples	Italy
Sinisi Leonardo	NEUROLOGY UNIT AND MS CENTER, ASL NAPOLI 1 CENTRO, NAPLES, ITALY	Italy
Sireci Francesca	UOC Neurologia- centro SM Reggio Emilia- AUSL-IRCCS RE	Italy
Sola Patrizia	Neurology Unit, Ospedale Civile, Azienda Ospedaliero-Universitaria di Modena, Modena, Italy	Italy
Solaro Claudio	Dept. of Rehabilitation, CRRF "Mons. Luigi Novarese", Moncrivello, Italy	Italy
Sormani Maria Pia	1. Department of Health Sciences, University of Genoa, Genoa, Italy.	Italy
	2. IRCCS Ospedale Policlinico San Martino, Genoa, Italy.	Italy
Sotgiu Stefano	Dipartimento di Scienze Mediche, Chirurgiche e Sperimentali - Università di Sassari	Italy
Sparaco Maddalena	Department of Advanced Medical and Surgical Sciences, University of Campania Luigi Vanvitelli, 80138 Naples, Italy;	Italy
Stromillo Maria Laura	Department of Medicine, Surgery and Neuroscience, University of Siena, Italy	Italy
Strumia Silvia	MS center, Neurology Unit, Morgagni-Pierantoni Hospital, Forlì	Italy
Susani Emanuela Laura	ASST GOM NIGUARDA, DIPARTIMENTO NEUROSCIENZE	Italy
Tabiadon Giulietta	Multiple Sclerosis Outpt.Clinic, Clinical Neurology and Stroke Unit Dep., Central Country Hospital, Bolzano, Italy	Italy
Teatini Francesco	Multiple Sclerosis Outpt.Clinic, Clinical Neurology and Stroke Unit Dep., Central Country Hospital, Bolzano, Italy	Italy
Tedeschi Gioacchino	Department of Advanced Medical and Surgical Sciences, University of Campania, Naples, Italy.	Italy
Tomassini Valentina	1. Institute for Advanced Biomedical Technologies (ITAB), Department of Neurosciences, Imaging and Clinical Sciences, University G. d'Annunzio of Chieti-Pescara, Chieti, Italy	Italy
	2. MS Centre, Department of Clinical Neurology, SS. Annunziata University Hospital, Chieti, Italy	Italy
Tonietti Simone	Centro Sclerosi Multipla Ospedale San Carlo Borromeo (Milano)	Italy
Torri Clerici Valentina	Centro Sclerosi Multipla, U.O Neurologia IV, Fondazione IRCCS Istituto Neurologico "Carlo Besta", Milano	Italy
Tortorella Carla	Dept Neurosciences, San Camillo Forlanini Hospital - Rome	Italy
Toscano Simona	Dipartimento Scienze Mediche e Chirurgiche e Tecnologie Avanzate, GF Ingrassia, Università di Catania; Centro Sclerosi Multipla Policlinico "G Rodolico"- San Marco, Università di Catania	Italy
Totaro Rocco	Demyelinating Disease Center, San Salvatore Hospital, L'Aquila	Italy
Trojano Maria	Department of Basic Medical Sciences, Neurosciences and Sense Organs - University of Bari Aldo Moro	Italy
Trotta Maria	Ospedale Annunziata (Cosenza)	Italy
Turano Gabriella	Department of Neurology, Regina Montis Regalis Hospital, Mondovì (CN)	Italy
Ulivelli Monica	Department of Medicine, Surgery and Neuroscience, University of Siena, Siena, Italy	Italy
Valentino Manzo	Neurological Clinic and Stroke Unit "A. Cardarelli Hospital", Naples, Italy;	Italy
Vaula Giovanna	Neurology II, AOU Città della Salute e della Scienza -Turin	Italy
Vecchio Domizia	Neurology Unit, Maggiore della Carità Hospital, Department of Translational Medicine, University of Piemonte Orientale, Novara, Italy	Italy
Vercellino Marco	MS Center, Department of Neuroscience, City of Health and Science University Hospital of Turin, Turin, Italy	Italy
Verrengia Elena Pinuccia	Centro Sclerosi Multipla, Ospedale di Legnano, ASST OVEST MI, Italia	Italy
Vianello Marika	UO Neurologia, Treviso	Italy
Virgilio Eleonora	Neurology Unit, Maggiore della Carità Hospital, Department of Translational Medicine, University of Piemonte Orientale, Novara, Italy	Italy
Vitetta Francesca	Neurology Unit, Ospedale Civile, Azienda Ospedaliero-Universitaria di Modena, Modena, Italy	Italy
Vollaro Stefano	Emergency Department, Guglielmo da Saliceto Hospital, Piacenza, Italy	Italy
Zaffaroni Mauro	Centro Sclerosi Multipla Ospedale di Gallarate, ASST della Valle Olona	Italy
Zampolini Mauro	Neurologia, Ospedale San Giovanni, Foligno	Italy
Zarbo Ignazio Roberto	Dipartimento di Scienze Mediche, Chirurgiche e Sperimentali - Università di Sassari	Italy
Zito Antonio	IRCCS Mondino Foundation, Pavia	Italy
Zuliani Luigi	Department of Neurology - Ospedale San Bortolo - AULSS8 Berica Vicenza	Italy
Jerome Grimaud	CH Chartres	France
Francois Sellal	CH Colmar	France
Guido Ahle	CH Colmar	France
Jean Christophe Seghezzi	CH Compiegne	France
Pierre Yves Garcia	CH Compiegne	France
Sarah Coulette	CH Gonesse	France
Eric Manchon	CH Gonesse	France

Arnaud Gagnol	CH Libourne	France
Philippe Kerschen	CH Luxembourg	France
Jérémie Papassin	CH Metropole Savoie	France
Fayçal Derouiche	CH Mulhouse	France
Mathilde Goudot	CH Mulhouse	France
Ombline Fagniez	CH Poissy	France
Olivier Heinzlef	CH Poissy	France
Marc Coustans	CH Quimper	France
Camille Rizzato	CH Vannes	France
Dorothee Videt	CH Vannes	France
Jennifer Aboab	CHNO 15-20	France
Maud Pallix-Guyot	CHR Orléans	France
Aurore Jourdain	CHRU Brest	France
Helene Zephir	CHRU Lille	France
Patrick Vermersch	CHRU Lille	France
Julie Boucher	CHRU Lille	France
Sophie Pittion	CHRU Nancy	France
Marc Debouverie	CHRU Nancy	France
Guillaume Mathey	CHRU Nancy	France
Jerome De Seze	CHRU Strasbourg	France
Kevin Bigaut	CHRU Strasbourg	France
Laurent Kremer	CHRU Strasbourg	France
Marie-celine Fleury	CHRU Strasbourg	France
Nicolas Collongues	CHRU Strasbourg	France
Eric Berger	CHU Besançon	France
Cecile Dulau-Metras	CHU Bordeaux	France
Aurélie Ruet	CHU Bordeaux	France
Jean-Christophe Ouallet	CHU Bordeaux	France
Bruno Brochet	CHU Bordeaux	France
Nathalie Derache	CHU Caen	France
Gilles Defer	CHU Caen	France
Pierre Branger	CHU Caen	France
Xavier Moisset	CHU Clermont Ferrand	France
Frédéric Taithe	CHU Clermont Ferrand	France
Pierre Clavelou	CHU Clermont Ferrand	France
Dominique Audry	CHU Dijon	France
Agnes Fromont	CHU Dijon	France
Thibault Moreau	CHU Dijon	France
Karine Mougine	CHU Dijon	France
Olivier Casez	CHU Grenoble	France
Catalina Coclitu	CHU Grenoble	France
Mathieu Vaillant	CHU Grenoble	France
Dalia Dimitri	CHU Kremlin Bicetre	France
Kumaran Deiva	CHU Kremlin Bicetre	France
Dan Buch	CHU Lariboisière	France
Alexis Montcuquet	CHU Limoges	France
Sandra Vukusic	CHU Lyon	France
Romain Marignier	CHU Lyon	France
Codjia Pekes	CHU Lyon	France
Iuliana Ionescu	CHU Lyon	France
Françoise Durand-Dubief	CHU Lyon	France
Julie Pique	CHU Lyon	France
Géraldine Androdias-Condemine	CHU Lyon	France
Audrey Rico	CHU Marseille	France
Adil Maarouf	CHU Marseille	France
Sarah Demortière	CHU Marseille	France
Clémence Boutiere	CHU Marseille	France
Bertrand Audoin	CHU Marseille	France
Jean Pelletier	CHU Marseille	France
Clarisse CARRA-DALLIERE	CHU Montpellier	France
Pierre Labauge	CHU Montpellier	France
Xavier Ayrignac	CHU Montpellier	France
David Laplaud	CHU Nantes	France
Flora Lejeune	CHU Nantes	France
Sandrine Wiertlewski	CHU Nantes	France
Christine Lebrun-Frenay	CHU Nice	France
Saskia Bresch	CHU Nice	France
Mikael Cohen	CHU Nice	France
Eric Thouvenot	CHU Nimes	France
Giovanni Castelnovo	CHU Nimes	France
Ayman Tourbah	CHU Paris IdF Ouest	France
Jean-Philippe Neau	CHU Poitiers	France
Nicolas Maubeuge	CHU Poitiers	France
Gilles Edan	CHU Rennes	France
Laure Michel	CHU Rennes	France
Emmanuelle Le Page	CHU Rennes	France
Anne Kerbrat	CHU Rennes	France
Bertrand Bourre	CHU Rouen	France
Damien Biotti	CHU Toulouse	France
Jonathan Ciron	CHU Toulouse	France
Adrien Delourme	CHU Toulouse	France
Anne-Marie Guennoc	CHU Tours	France
Aude Maurousset	CHU Tours	France
Stéphane Beltran	CHU Tours	France
Lucile Gleyze	CHU Tours	France
Marie Théaudin	CHU Vaudois (Suisse)	France
Renaud Du Pasquier	CHU Vaudois (Suisse)	France
Caroline Pot	CHU Vaudois (Suisse)	France
Arnaud Kwiatkowski	GHIC Lille	France
Patrick Hautecoeur	GHIC Lille	France
Julien Poupart	GHIC Lille	France
Magalie Rabin	GHT Atlantique	France
Maya Tchikviladze	Hôpital Foch	France
Christine Clerc	Neurologue Libéral	France
Hervé Fayolle	Neurologue Libéral	France
Evelyne Planque	Neurologue Libéral	France
Laurent Guilloton	Neurologue Libéral	France
Alain Créange	Paris Créteil HM	France
Abir Wahab	Paris Créteil HM	France
Mickaël Zedet	Paris Créteil HM	France
Samar S. Ayache	Paris Créteil HM	France
Caroline Bensa-Koscher	Paris FOR	France
Antoine Gueguen	Paris FOR	France
Romain Deschamps	Paris FOR	France
Olivier Gout	Paris FOR	France
Céline Louapre	Paris PSL	France
Ariële Azoulay-Cayla	Paris PSL	France
Caroline PAPEIX	Paris PSL	France
Edouard Januel	Paris PSL	France
Jean Christophe Corvol	Paris PSL	France
Raphael De Paz	Paris PSL	France
Catherine Lubetzki	Paris PSL	France
Elisabeth Maillart	Paris PSL	France
Michella Ibrahim	Paris PSL	France
Rana Assouad	Paris PSL	France
Salimata Gassama	Paris PSL	France
Sinead Zeidan	Paris PSL	France
Vito AG Ricigliano	Paris PSL	France
Thomas Roux	Paris PSL	France
Ysoline Beigneux	Paris PSL	France
Amandine Bordet	Paris PSL	France
Yanica Mathieu	Paris PSL	France
Véronique Marcaud	Paris Saint Joseph	France
Anne-Laure Dubessy	Paris SAT	France
Aurelian Ungureanu	Paris SAT	France
Benedetta Bodini	Paris SAT	France
Claire Giannesini	Paris SAT	France
Bruno Stankoff	Paris SAT	France
Jean Capron	Paris SAT	France
Frederique Le Breton	Paris Tenon	France
